# A QSAR Study of Matrix Metalloproteinases Type 2 (MMP-2) Inhibitors with Cinnamoyl Pyrrolidine Derivatives

**DOI:** 10.3797/scipharm.1112-21

**Published:** 2012-01-31

**Authors:** Eduardo Borges de Melo

**Affiliations:** Theoretical Medicinal and Environmental Chemistry Laboratory (LQMAT), Department of Pharmacy, Western Paraná State University (Unioeste), 2069 Universitária St, 8519110, CascaveI, PR, Brazil.

**Keywords:** Matrix metalloproteinases, MMP2, Gelatinases, Cancer, QSAR, OPS

## Abstract

A multivariate PLS-QSAR study with a data set of 31 cinnamoyl pyrrolidine derivatives described as type 2 matrix metalloproteinases (MMP-2) inhibitors is presented in this paper. The variable selection was performed with the Ordered Predictors Selection (OPS) algorithm. The PLS model presented six descriptors and three Latent Variables (LV) that cumulated 71.845% of variance. Leave-N-out (LNO) cross validation and y-randomization tests showed that the model presented robustness and no chance correlation, respectively. The descriptors indicated that MMP-2 inhibition depends mainly on the electronic properties of the compounds. The model obtained can be useful as a support tool in the design of new MMP-2 inhibitors.

## Introduction

The matrix metalloproteinases (MMPs) are a family of enzymes that are intimately involved in tissue remodeling. These zinc-containing endopeptidases consist of subsets of enzymes, and they are involved in the degradation of the extracellular matrix (ECM) that forms the connective material between cells and around tissues. In pathologic conditions an increase of MMP activity occurs, leading to tissue degradation [1].

**Fig. 1.**
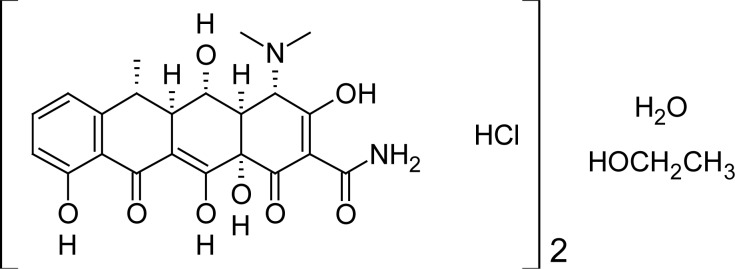
Structural formula of doxycycline hyclate (Periostat®, CollaGenex Pharmaceuticals).

Currently, about 27 MMPs are known. Their overexpression is associated with several diseases: cancer, cardiovascular diseases (including congestive heart failure), osteoarthritis, rheumatoid arthritis, chronic obstructive pulmonary disease, psoriasis, dermatitis, Alzheimer’s disease and periodontitis, among others [1, 2]. Thus, MMPs are currently an interesting target for drug design. However, despite the great amount of research, the tetracycline doxycycline (Fig. 1) is the only MMP inhibitor available in therapeutics. This longer-acting antibiotic also presents a weak inhibition of collagenases (MMPs-1, 8 and 13), and it is currently marketed for clinical treatment of chronic periodontal disease [3–5].

Among the MMPs, MMP2 and MMP9 are named gelatinases. These enzymes are able to degrade a broad range of matrix substrates, including gelatin, type IV collagen of basal laminae, as well as other nonhelical collagen domains and proteins, such as fibronectin and laminin, that constitute cellular connective tissue and are strongly involved in both normal and pathological tissue remodeling [1, 6]. The overexpression of this subclass, especially MMP2, is found to be strongly correlated to an aggressive malignant phenotype, and it presents poor prognosis for several types of aggressive cancer, such as ovarian, lung, breast, bladder and gastric cancers [6–8]. Thus, MMP2 inhibitors have been studied as a target for anticancer drug design.

**Fig. 2.**
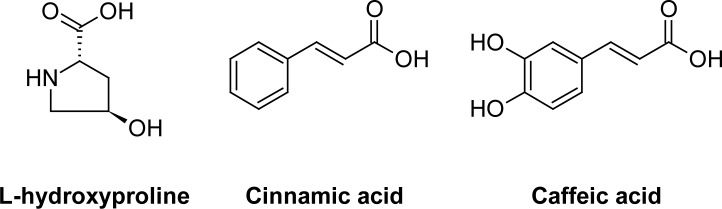
Structures of L-hydroxyproline, cinnamic acid and caffeic acid.

Quantitative structure-activity relationship (QSAR) describes how a given biological activity can vary as a function of molecular descriptors derived from the chemical structure of a set of molecules. A model containing those calculated descriptors can be used to predict responses from new compounds, constituting an important tool to support the synthesis of new drugs [9, 10]. Thus, considering the continuous need for new anticancer drugs, a QSAR study based on 31 cinnamoyl pyrrolidine derivatives (Table 1) synthesized and assayed by Zhang et al. [8] was carried out. The dataset was obtained through a hybridization approach between the L-hydroxyproline scaffold, the MMPs substrate, the cinnamic acid, an inhibitor of the A5491 human lung gland cancer, and the caffeic acid, an MMP-2 inhibitor (Fig. 2). The aim was obtaining a mathematical model that could be used for prediction of the inhibitory potency of new cinnamoyl pyrrolidine derivatives against MMP-2.

## Results and Discussion

The study was carried out using the QSAR Modeling [11]. The variable selection with the Ordered Predictors Selection (OPS) algorithm [12–15] generated a model based on three Latent Variables (LV) that cumulate 71.845% of variance (LV1: 18.043%; LV2: 31.298%; LV3: 22.504%). These LV derivate from six selected descriptors: SOFT (softness), EEig02r (eigenvalue 02 from edge adjacent matrix weighted by resonance integrals), α_xx_ (the component vector to the overall polarizability in the *x*-axis), q10NBO (partial charge of the atom #10 calculated through Natural Bond Orbitals approach), q2NBO (partial charge of the atom #2 calculated through Natural Bond Orbitals approach) and SsssN(oth) (E-state index for amino group attached to functional groups not aliphatic or aromatic). The values of each descriptor are available in the Supporting Information, Table S1. The standardized regression coefficients are −0.549 for EEig02r, 0.545 for SOFT, 0.377 for α_xx_, 0.238 for q10NBO, 0.250 for q1NBO, and −0.314 for SsssN(oth). According to Wold [16], regression coefficients larger than about half the maximum regression coefficient value indicate that the descriptor is significant for the PLS-QSAR model. Thus, the reference value is 0.274. The coefficients of q2NBO and q10NBO are lower than this value, but its removal decreases the statistical quality of the model. Thus, these descriptors can be considered important for the model. In addition, the maximum difference is only 0.036 units, which is very low. Thus, both descriptors were maintained in the model.

**Fig. 3.**
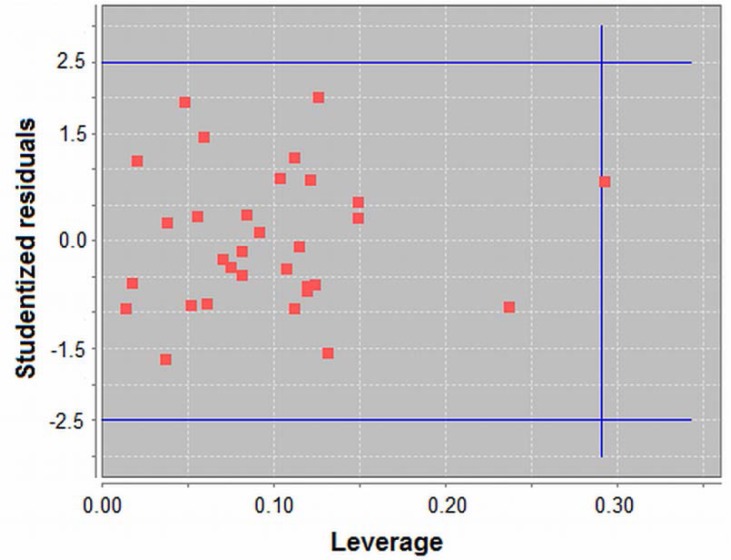
Outlier detection plot. The figure was built in QSAR Modeling [11].

Fig. 3 shows the studentized residuals (σ) versus the leverage samples plot, and it was used for the identification of outliers. No compound presented residuals higher than 2.5xσ. Only one compound presented leverage higher than the leverage cutoff line, but it can be considered acceptable [17]. Therefore, the model can be considered free of outliers, something which guarantees the maximum possible representation in terms of structure and range of inhibitory activity for the dataset under study.

The model (Equation I) explains 78.324% (*R^2^*=0.783) and predicts 61.844% (*Q^2^_LOO_*=0.618) of variance. The predicted values in the cross-validation step and the residuals are available in the Supporting Information, Table S2. The difference between the values of *R^2^* and *Q^2^*_LOO_ was 0.165 units. A large difference between *R^2^* and *Q^2^*_LOO_ exceeding 0.2–0.3 is a clear indication that the model suffers from overfitting [18]. Thus, this difference may be considered acceptable. The *F* value (32.521) was higher than the corresponding tabled value (*p*=3 and *n*-*p*-1=27) with a 95% confidence interval (α=0.05). The value of *PRESS*_val_ was smaller than *SS_y_*, another indicator of the statistical significance of the prediction [16].

Eq. 1. $$\begin{array}{l} \begin{array}{l} p{\text{IC}}_{50}=0.394(\text{SOFT})-2.198(\text{EEig}02\text{r})+0.014(\alpha_{\text{xx}})+80.105(\text{q}10\text{NBO})+\\ 11.339(\text{q}2\text{NBO})-9.218(\text{SsssN}(\text{oth}))+64.222 \end{array}\\ \begin{array}{l} n=31;\;\;\;\;R^{2}=0.783;\;\;\;\mathrm{SEC}=0.276;\;\;\;F_{\left(3,27\right)}=32.521\;\;\;(cF=2.960);\;\;\;\;Q^{2}{}_{\text{LOO}}=0.618;\\ \mathrm{SEV}=0.342;{\mathrm{PRESS}}_{\text{val}}=3.621({\mathrm{SS}}_{y}=9.491). \end{array} \end{array}$$

**Fig. 4.**
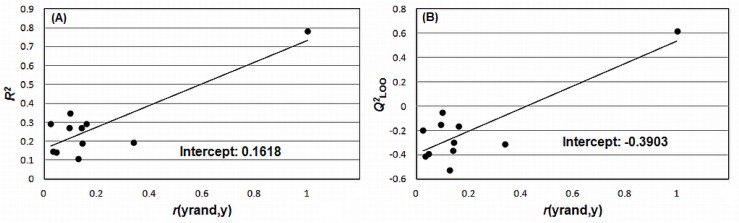
Results of y-randomization test (A and B). The “*r*(yrand,y)” values in the x-axis are presented in absolute values. Figure built from the results generated in QSAR Modeling [11].

**Fig. 5.**
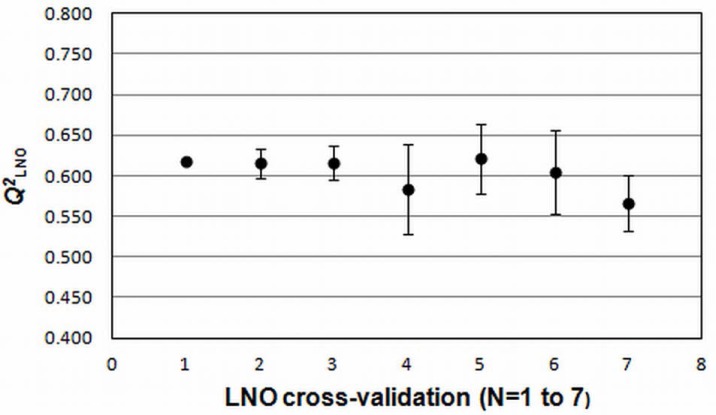
Results of LNO cross validation. The bars in the graphic represent standard deviations with regard to six tests for each “*N*” value. Figure built from the results generated in QSAR Modeling [11].

The results obtained from y-randomization [19] analysis and LNO cross-validation [20] are available in Figs. 4 and 5. The y-randomization aids in verifying the possibility that the explained and predicted variances are due to chance correlation [19]. It can be observed that the results obtained for all randomized models have a bad quality when compared to the original model, because the intercepts are within the acceptable values recommended in literature, i.e., below 0.3 (Fig. 4A) and 0.05 (Fig. 4B). These results indicate that the variance explained by the model was not due to chance correlation.

LNO cross-validation (Fig. 5) employs smaller training sets than the LOO cross-validation, and it can be repeated several times, because of the large number of combinations that rise when more than one compound is left out from the training set, once at a time. A QSAR model can be considered robust when the average values of *Q^2^*_LNO_ are relatively high and close to *Q^2^*_LOO_ [19]. The model obtained in this study has an average *Q^2^*_LNO_ (0.604), only 0.014 units lower than *Q^2^*_LOO_. The standard deviation for each “*N*” (performed in hexaplicate) value is small, with the maximum of 0.055 for *Q^2^_L4O_*.

Some studies show that only externally validated models may be considered realistic and applicable for drug design [21–24]. The real model (II) was obtained after the split of data in training (*n*=26) and test (*n*=5) sets. The standardized regression coefficients of each descriptor are −0.579 for EEig01x, 0.599 for SOFT, 0.362 for α_xx_, 0.149 for q10NBO, 0.322 for q1NBO, and −0.278 for SsssN(oth). The model (II) has statistical parameters similar to those for the auxiliary model (i.e., Eq. 1). Therefore, they can be considered equivalent and can be used in the external validation.

Eq. 2. $$\begin{array}{l} \begin{array}{l} p{\text{IC}}_{50}=0.450(\text{SOFT})-2.293(\text{EEig}01\text{x})+0.013(\alpha_{\text{xx}})+61.930(\text{q}10\text{NBO})+\\ 14.508(\text{q}2\text{NBO})-8.637(\text{SsssN}(\text{oth}))+55.156 \end{array}\\ \begin{array}{l} n=26;\;\;\;\;R^{2}=0.809;\;\;\;\mathrm{SEC}=0.264;\;\;\;F_{\left(3,22\right)}=31.089\;\;\;(cF=3.049);\;\;\;\;Q^{2}{}_{\text{LOO}}=0.626;\\ \mathrm{SEV}=0.340;{\mathrm{PRESS}}_{\text{val}}=3.000({\mathrm{SS}}_{y}=8.026). \end{array} \end{array}$$

Results obtained for the external validation (Table 2) show that the model has high external prediction power, considering the proposed limits. *R^2^*_pred_, tool used as a measure of the model’s external predictive power, was higher than the adopted threshold (*R^2^*_pred_ = 0.641 > 0.5), and the associated error (*SEP*) with this parameter may be considered low. The Golbraikh-Tropsha statistics [25, 26] aid to confirm the prediction power of the model. Both values of *k* and *k’* and the relation **|***R^2^_0_*−*R’^2^_0_***|** are within acceptable ranges (0.85 ≤ *x* ≤ 1.15, where *x* = *k* or *k’,* and **|***R^2^_0_*−*R’^2^_0_*| < 0.3).

**Fig. 6.**
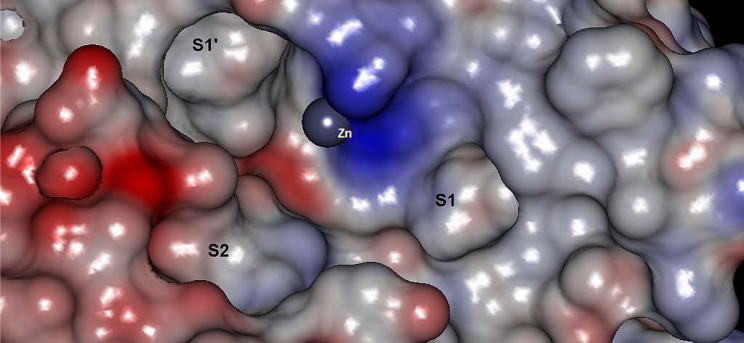
Representation of the binding site of metalloproteinases, highlighting the most important points (sites S1, S1’, S2, and Zn^+2^). The structure used corresponds to MMP-3 (stromelysin-1), PDB 1D7X [28]. The figure was built in Accelrys Discovery Studio Visualizer 2.5 [29].

It can be observed that the obtained model has reasonable internal and external quality. However, it is always desirable to obtain a model that is able to relate the physicochemical properties represented by the selected molecular descriptors to the action mechanism of the system under study [27]. Zhang et al. [8] described the experimental structure-activity relationships of the data set, highlighting the importance of heteroatoms (especially the hydroxil group) to form hydrogen bonds, and π electrons to facilitate interactions with hydrophobic regions of the receptor, and a slight decrease in inhibitory potency with the addition of methoxyl to R_1_ and R_2_. Furthermore, a docking study indicated that the ester carbonyl (atom #20) could bind with the zinc located in the active site, the lateral chain represented in this paper by R_3_ bind with the S1’ cavity, and the lateral chain attached to the nitrogen bind with the S1 cavity. A representation of the metalloproteinases active site [28, 29] is presented in Fig. 6.

**Fig. 7.**
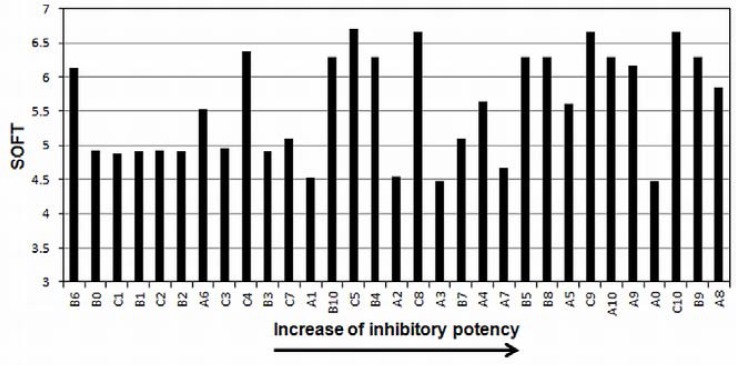
Histogram presenting the SOFT of dataset in relation to MMP-2 inhibitory potency.

The SOFT, a quantum chemical descriptor, was calculated using the relation SOFT=1/GAP, where GAP is the difference between the energies (calculated at B3LYP/6-311(d,p) theory level) of lowest unoccupied molecular orbital and highest occupied molecular orbital (E_LUMO_−E_HOMO_). These molecular descriptors are known to be related to molecular reactivity. Generally, softer molecules are more reactive [26, 30]. As the SOFT coefficient is positively correlated to *p*IC_50_, this indicates that derivatives with high value for this descriptor will react more easily. The histogram presented in Fig. 7 shows exactly this trend: considering the 16 most active compounds, only four (**A2**, **A3**, **A7**, and **A0**) have SOFT < 5. The compounds found among the most active have a greater tendency to present many heteroatoms (oxygen and chlorine) and π electrons in the substituent R_3_, in agreement with the experimental structure-activity relationships discussed by Zhang et al. [8], probably by facilitating the interaction with the enzyme via hydrogen and hydrophobic bonds. Thus, similar to what was proposed by Liu et al. for a set of α-glucosidase inhibitors [30], the inhibitory activity would be expected to be improved by introducing more heteroatoms and electrons π in the structure of new derivatives.

The EEig02r, which presents a negative coefficient, is an edge adjacency index, a topological descriptor derived from the edge adjacency matrix, also called bond matrix, which encodes the connectivity between graph edges [26, 31]. In this approach, as in many other graph theoretical representations of chemical structures, the vertices of the molecular graph represent atoms and edges represent bonds in molecules. The edge adjacency index with this weighting scheme is sensitive to the presence of heteroatoms and multiple bonds in the molecule [26]. This class of descriptors can be weighted by several different atomic properties. The most interesting aspect of the presence of a weighted-resonance index in the model is that this weighting scheme turns the descriptor more sensitive to the presence of heteroatoms and multiple bonds in the molecule [26]. So, its selection by OPS algorithm may be, again, related to the importance of heteroatoms and π electrons in the R_3_ substituent.

**Fig. 8.**
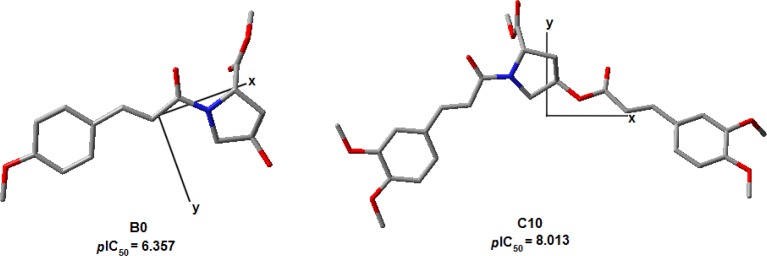
Cartesian axes’ representation for compounds **B0** and **C10**. The *z*-axis is located perpendicular to the plane of projection.

The α_xx_, calculated in the Marvin 4.1.8 [32] through a method based on the empiric model proposed by Miller and Savchik [33], describes the ability of a molecule to be polarized in the X Cartesian axis. The signal of the coefficient is positive, indicating that the improvement of the polarization in this plane is favorable to the activity. In Fig. 8 it is possible to see that the *x*-axis always crosses the frontal region of the structures. The size of R_3_ substituent causes a slight shift in the position of the axis, as it can be seen in the compounds **C0** (low potent) and **C10** (high potent). This information can be related to the interpretation proposed for the SOFT, since the presence of a greater number of heteroatoms and π electrons in R_3_ increase the polarization of this Cartesian axis.

The q2NBO and q10NBO are atomic charges descriptors calculated using the Natural Bond Orbital (NBO) theory. The charges measure the extent of electronic density localization in a molecule. Negative q*_n_* values mean that there is excess electronic charge in the atom while positive values mean that the atom is electron-deficient [26]. It is possible to observe that the charge of atom #2 undergoes a slight increase in electron density (see Supporting Information, Table S1) in subsets **B** and **C**, probably due to an electron donor effect resulting from the insertion of the methoxyl at positions R_1_ and R_2_. This effect was more pronounced in the subset **B** (only R_2_ substituent) than in the subset **C** (substituents at R_1_ and R_2_). Interestingly, the compounds of subset **A** are generally more potent than their corresponding in subsets **B** and **C**, which have, in general, higher electron densities in the atom #1. It can be proposed, since the sign of its coefficient is positive, that an electron donor effect caused by the insertion of the methoxyl in the aromatic ring decreases its electron density, hampering the interaction of this group with the S1 site of MMP-2. This same effect can be observed, in a less pronounced manner, in the atom #10, the nitrogen of pyrrolidine ring, since the descriptor q10NBO also has a positive coefficient.

The SsssN(oth) is an atom type E-state (electrotopological state) index, and it also corresponds to the nitrogen from the pyrrolidine ring. The E-state formalism considers that each atom or bond has an intrinsic state, which is disturbed by every other atom or bond in the molecule. This state encodes information about the electronic distribution (as a variation caused by all other atoms) and topological aspects (major/minor accessibility of atoms and bonds to the external environment), and how such information can influence intermolecular interactions [26, 34]. Since this descriptor is also related to the atom #10, this indicates that, although the most important point of structural variation for the activity is the R_3_ substituent, other parts of the molecule also influence the activity. The pyrrolidine nitrogen, for example, is close to the ester carbonyl side chain, the binding point with the zinc atom located in the active site of MMP-2. The negative coefficient indicates that the decrease of this descriptor is favorable to the activity. Among the dataset, the lowest SsssN(oth) values are in the A subset (Supporting Information, Table S1). This subset has no substituents in R_1_ and R_2_ (Table 1). Thus, it may indicate that these substitutions also affect the intrinsic value of nitrogen, as well as the partial charge descriptor q10NBO, influencing the interactions that this part of the molecule can have with the binding site of MMP-2.

Interestingly, the three most important descriptors (EEig02r, SOFT and α_xx_), considering the standardized coefficients of the real model (Eq. 2), are related exactly to the R_3_ substituents, the main point of structural variation in the dataset, and it is therefore primarily responsible for the variation in inhibitory potency. This result strengthens the importance of hydrogen and hydrophobic bonds to S1' binding site of MMP-2, and demonstrates how the manipulation of this characteristic in structurally related compounds can be useful in the design of new cinnamoyl pyrrolidine derivatives able to inhibit MMP-2.

## Conclusion

The model obtained using the OPS, an algorithm for variable selection, showed a statistically significant internal and external prediction power. In addition, the LNO cross-validation shows the model is robust, and in the y-randomization test it shows the model does not present chance correlation. The selected descriptors suggest that the presence of heteroatoms, especially, and π electrons in the R_3_ substituent can be important for the binding of compounds to the regions S1’ of the binding site of MMP-2, but the handling of electronic distribution in the side chain attached to the pyrrolidinic nitrogen, which binds to the S1 site, can also be exploited for the design of new active derivatives. The manipulation of these features can assist in obtaining new lead compounds that can be useful for developing new drugs used in the chemotherapy for treating aggressive cancers.

## Experimental

### Molecular Modeling

Three-dimensional structures were built using HyperChem 7 [35] from the structure ZINC40405643, obtained in the ZINC Database (http://zinc.docking.org) [36]. Calculations of MM+ force field were carried out using the same software. The most stable conformations obtained were further optimized at AM1 semi-empirical quantum mechanical method, followed by Hartree-Fock level (HF/6-31G(d)) and Density Functional Theory (DFT) level (B3LYP/6-311G(d,p)) using Gaussian 09 [37]. The DFT/B3LYP was chosen as method for obtaining the geometries and electronic properties because it leads to quite satisfactory results in the analysis with such aims [9, 10].

### Molecular descriptors

The SMILES strings [38] of each compound were used to obtain E-state indices in the Parameter Client [39]. The optimized geometries were used to obtain, in the Dragon 3.0 Web Version [31], the following classes of descriptors: constitutional descriptors, functional groups counts, charge descriptors, molecular properties, walk and path counts, information indices, edge adjacency indices, topological charge indices, topological descriptors, connectivity indices, 2D autocorrelations, Burden eigenvalues, and eigenvalue-based indices. The optimized geometries were also used to obtain the electronic descriptors in the Gauss View 5 [40]. Partial charges of the basic structure were calculated by means of two approaches: Mulliken Charges and Natural Bond Orders [41]. In the Marvin 4.1.8 [32], it was obtained the molecular polarizability (α) and the respective vectorial components (α_xx_, α_yy_ and α_zz_). After removal of missing, invariants, and quasi-invariants descriptors calculated in the Dragon 3.0, a total of 439 molecular descriptors were available for use.

### Mathematical method

The partial least squares (PLS), a classical chemometric method, was employed to explore the quantitative relationships between the training set and MMP-2 inhibition. In this calibration method, LV are obtained including the dependent variable (in this case, *p*IC_50_) in the analysis in such a way that the covariance between the projection of the samples in the new axis system (also orthogonal) and the dependent variable is maximized [42, 43]. For this, descriptors should be preprocessed using the autoscaling scheme (columnwise mean-centered and scaled to unity variance). Thus, they can be compared to each other on the same scale.

### Variable selection

The step of variable selection in a QSAR study is a way to identify reduced subsets of descriptors that in fact reproduce the observed values of a biological activity, i.e. those that are the most useful to obtain a more accurate prediction model. The use of a good variable selection method helps to obtain the subset to reach an optimal mathematical equation for the prediction of the activity under study and, therefore, simple, robust, and more easily interpretable models [44, 45]. In this study, a two-step procedure was employed: (i) the 439 original descriptors were reduced to 81 by eliminating those that presented the absolute value of Pearson’s correlation coefficient (|*r*|) with pIC_50_ lower than 0.3; and (ii) the ordered predictor selection (OPS) algorithm [12–15] was used to select the most important descriptors. OPS is able to build PLS models by rearranging the columns of the matrix in such a way that the most important descriptors, classified according to an informative vector (available options: correlation vector, regression vector and an element-wise product between both), are placed in the first columns. Then, successive PLS regressions are performed with an increasing number of descriptors to find the best model. In this work, the three informative vectors were used. The best models were classified in descending order of statistical quality according to their coefficient of determination of leave-one-out cross validation (*Q^2^*_LOO_) or standard error of cross validation (*SEV*) values. OPS is implemented in QSAR Modeling [11], a free JAVA-based software developed by the courtesy of the Theoretical and Applied Chemometrics Laboratory’s research group (http://lqta.iqm.unicamp.br).

### Model validation

Several statistical tools (see Supporting Information) are suggested in literature for validation of QSAR models. For the internal quality, the adopted parameters were the coefficient of multiple determination of calibration (*R^2^*), standard error of calibration (*SEC*), *F*-ratio test with a 95% confidence interval (*F*, α=0.05) *Q^2^*_LOO_, *SEV* and predictive residual sum of squares of validation (*PRESS*_val_) [18]. The adopted limits are *R^2^* > 0.6 and *Q^2^*_LOO_ > 0.5. *SEC* and *SEV* values should be as low as possible. For *PRESS*_val_, values should be lower than the sum of squares of the response values (SSy) [19]. *F-test* value should be higher than the tabled *F* value (*F_p_*_,_*_n_*_−_*_p_*_−1_, where *n* is the number of compounds and *p* is the number of LV) and the higher the difference between them, the more statistically significant is the model [46].

The robustness of the model was examined through leave-*N*-out (LNO) cross validation, with *N*=1 to 7. This test was repeated three times for each “*N*” value. All rows from the data matrix and respective y values were randomized in each step of LNO process. It is expected that the average value of each *Q^2^*_LNO_ would be close to *Q^2^*_LOO_ (coefficient of multiple determination of leave-one-out cross validation) with standard deviations close to zero [21]. The possibility of chance correlation was tested using y-randomization test, where only the y vector (*p*IC_50_) was scrambled 10 times. The approach suggested by Eriksson et al. [20], based on the |*r*| between the original vector y and the randomized vectors y, was used to quantify chance correlation. In this approach, two regression lines are built using these correlation coefficients (*x*-axis) and the *R^2^* and *Q^2^*_LOO_ values (*y*-axis). The intercepts of the equations obtained in the linear regression should be lower than 0.3 for *R^2^* and 0.05 for *Q^2^*_LOO_.

Once internally validated, the data set was split into training set (*n*=26) and test set (*n*=5), generating the real model [18]. The test set was selected manually, in such a way that the entire range of *p*IC_50_ (6.25 to 8.208, 1.958 logarithmic units) and the structural variations of the data set were well represented. A dendrogram obtained for the complete data set by Hierarchical Cluster Analysis (HCA) [47] (Supporting Information, Fig. S1) aid to confirm that the selected compounds are suitable as test set. Thus, a structurally representative test set could be formed by the compounds **B2** (*p*IC_50_=6.553), **C4** (*p*IC_50_=6.696), **C5** (*p*IC_50_=6.952), **C9** (*p*IC_50_=7.542), and **A0** (*p*IC_50_=7.951). The HCA analysis are performed in Pirouette 4 [48].

The parameter coefficient for multiple determination of prediction (*R^2^_pred_*) and standard error of external prediction (*SEP*) was used as a measure of the predictive power of a QSAR model. The recommended limit is *R^2^*_pred_ > 0.5 [49], and *SEP* values also should be as low as possible. However, this is not enough to guarantee that the model is really predictive. It is also recommended to check: (i) the slopes *k* or *k’* of the linear regression lines between the observed activity (*y_i_*) and the predicted activity in the external validation (*ŷ_ei_*), where the slopes should be 0.85 ≤ *x* ≤ 1.15 (*x* = *k* or *k’*); and (ii) the absolute value of the difference between the coefficients of multiple determination, *R^2^_0_* and *R’^2^_0_*, smaller than 0.3 [26, 27].

## Figures and Tables

**Tab. 1.** Selected data set of cinnamoyl pyrrolidine derivatives and their respective inhibition potencies against MMP-2.

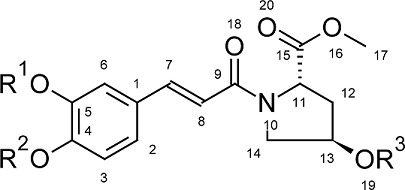					

**Compound^a^**	**R^1^**	**R^2^**	**R^3^**	**IC_50_ (nM)**	***p*IC_50_^b^**
**A0**	H	H	H	11.2	7.951
**A1**	H	H	CH_3_CO	128.4	6.891
**A2**	H	H	CH_3_CH_2_CO	98.1	7.008
**A3**	H	H	CH_3_CH_2_CH_2_CO	85.6	7.068
**A4**	H	H	PhCO	52.4	7.281
**A5**	H	H	*p*-Cl-PhCO	31.8	7.498
**A6**	H	H	2,3,4-(OCH_3_)_3_-PhCO	259.5	6.586
**A7**	H	H	PhCH_2_CH_2_CO	43.6	7.361
**A8**	H	H	PhCH=CHCO	5.2	8.284
**A9**	H	H	*p*-CH_3_O-Ph-CH=CHCO	12.3	7.910
**A10**	H	H	3,4-(OCH_3_)_2_-PhCH=CHCO	13.1	7.883
**B0**	H	CH_3_	H	439.8	6.357
**B1**	H	CH_3_	CH_3_CO	316.4	6.500
**B2**	H	CH_3_	CH_3_CH_2_CO	280.2	6.553
**B3**	H	CH_3_	CH_3_CH_2_CH_2_CO	195	6.710
**B4**	H	CH_3_	PhCO	109.9	6.959
**B5**	H	CH_3_	*p*-Cl-PhCO	42.8	7.369
**B6**	H	CH_3_	2,3,4-(OCH_3_)_3_-PhCO	562.6	6.250
**B7**	H	CH_3_	PhCH_2_CH_2_CO	73.4	7.134
**B8**	H	CH_3_	PhCH=CHCO	39.1	7.408
**B9**	H	CH_3_	*p*-CH_3_O-Ph-CH=CHCO	7.8	8.108
**B10**	H	CH_3_	3,4-(OCH_3_)_2_-PhCH=CHCO	121.3	6.916
**C1**	CH_3_	CH_3_	CH_3_CO	320.2	6.495
**C2**	CH_3_	CH_3_	CH_3_CH_2_CO	293.4	6.533
**C3**	CH_3_	CH_3_	CH_3_CH_2_CH_2_CO	221.1	6.655
**C4**	CH_3_	CH_3_	PhCO	201.2	6.696
**C5**	CH_3_	CH_3_	*p*-Cl-PhCO	111.8	6.952
**C7**	CH_3_	CH_3_	PhCH_2_CH_2_CO	168.3	6.774
**C8**	CH_3_	CH_3_	PhCH=CHCO	86.5	7.063
**C9**	CH_3_	CH_3_	*p*-CH_3_O-Ph-CH=CHCO	28.7	7.542
**C10**	CH_3_	CH_3_	3,4-(OCH_3_)_2_-PhCH=CHCO	9.7	8.013

a same identification used the original work [8];

b *p*IC_50_ = −log IC_50_.

**Tab. 2.** Results from external validation step performed through the real model (II).

**Compound**	***p*IC_50_ observed**	***p*IC_50_ predicted**	**Residuals**
**A0**	7.951	7.622	0.329
**B5**	7.369	6.954	0.415
**C2**	6.533	6.207	0.326
**C4**	6.696	7.069	−0.373
**C9**	7.542	7.601	−0.059

***R^2^*_pred_**	0.641		
***SEP***	0.325		
***k***	1.017		
***k’***	0.981		
**|*R*^2^_0_−*R*’^2^_0_|**	0.004		

## Supporting Information

Values of selected descriptors for each compound are available in Table S1. The results of leave-one-out cross-validation are available in Table S2. The dendrogram used to aid in the selection of test set is available in Figure S1. Statistics parameters and adopted limits for the evaluation of the quality of the QSAR model are also available as supporting information. These documents are available in the online version (Format: PDF, Size: < 0.1 MB): http://dx.doi.org/10.3797/scipharm.1112-21.
