# Supplementary material for: A QSAR Study of Matrix Metalloproteinases Type 2 (MMP-2) Inhibitors with Cinnamoyl Pyrrolidine Derivatives
Source: Sci Pharm. 2012 Jan 31;80(2):265–81. doi: 10.3797/scipharm.1112-21 (PMC3383210; doi:10.3797/scipharm.1112-21)
Supplement: Supplementary file 1 [file Scipharm-2012-80-265supportinginformation.pdf]

Scientia Pharmaceutica www.scipharm.at

Open Access

Supporting Information to

A QSAR Study of Matrix Metalloproteinases Type 2 (MMP-2) Inhibitors with Cinnamoyl Pyrrolidine Derivatives

Eduardo Borges DE MELO

Published in Sci Pharm. 2012; 80: 265–281 doi:10.3797/scipharm.1112-21

Available from: http://dx.doi.org/10.3797/scipharm.11121-21

© de Melo; licensee Österreichische Apotheker-Verlagsgesellschaft m. b. H., Vienna, Austria.

This is an Open Access article distributed under the terms of the Creative Commons Attribution License (http://creativecommons.org/licenses/by/3.0/), which permits unrestricted use, distribution, and reproduction in any medium, provided the original work is properly cited.

Table of Contents

Tab. S1. Values of the selected descriptors for the formulation of model.

Tab. S2. Results of leave-one-out (LOO) cross-validation.

Fig. S1. Dendrogram of the data set, with the test set compounds.

Statistical Parameters and Adopted Limits for the Evaluation of the Quality of the QSAR Model

Tab. S1. Values of the selected descriptors for the formulation of model.

Compound	EEig02r	SOFT	α_{xx}	q10NBO	q2NBO	SsssN(oth)
**A0**[a]	3.540	4.484	31.097	-0.470	-0.085	1.332
**A1**	3.542	4.525	35.841	-0.478	-0.081	1.341
**A2**	3.544	4.545	25.315	-0.475	-0.086	1.349
**A3**	3.544	4.484	26.605	-0.475	-0.085	1.336
**A4**	3.784	5.650	29.245	-0.476	-0.086	1.353
**A5**	3.804	5.618	30.306	-0.474	-0.085	1.368
**A6**	4.024	5.525	58.294	-0.475	-0.086	1.358
**A7**	3.633	4.673	48.537	-0.474	-0.085	1.344
**A8**	3.569	5.848	59.292	-0.474	-0.085	1.349
**A9**	3.645	6.173	63.647	-0.474	-0.085	1.358
**A10**	3.855	6.289	67.700	-0.474	-0.085	1.366
**B0**	3.635	4.926	33.170	-0.472	-0.112	1.381
**B1**	3.635	4.902	38.680	-0.475	-0.114	1.384
**B2**[a]	3.636	4.902	27.174	-0.477	-0.113	1.353
**B3**	3.636	4.902	45.619	-0.477	-0.113	1.370
**B4**	3.784	6.289	52.650	-0.477	-0.113	1.385
**B5**	3.804	6.289	57.117	-0.477	-0.114	1.374
**B6**	4.024	6.135	60.106	-0.476	-0.113	1.361
**B7**	3.642	5.102	53.945	-0.476	-0.113	1.330
**B8**	3.636	6.289	61.734	-0.476	-0.113	1.362
**B9**	3.646	6.289	65.864	-0.476	-0.112	1.371
**B10**	3.855	6.289	70.179	-0.475	-0.112	1.379
**C1**	3.855	4.878	43.205	-0.475	-0.097	1.395
**C2**	3.853	4.926	50.236	-0.477	-0.095	1.398
**C3**	3.853	4.950	53.002	-0.477	-0.095	1.366
**C4**[a]	3.853	6.369	44.454	-0.477	-0.084	1.383
**C5**[a]	3.853	6.711	62.776	-0.477	-0.096	1.398
**C7**	3.853	5.102	64.451	-0.477	-0.096	1.373
**C8**	3.853	6.667	67.106	-0.476	-0.095	1.388
**C9**[a]	3.853	6.667	71.482	-0.475	-0.095	1.374
**C10**	3.853	6.667	75.779	-0.475	-0.095	1.343

[a] test set.

Tab. S2. Results of leave-one-out (LOO) cross-validation.

Compound	*p*IC_{50} observed	*p*IC_{50} predicted	Residuals
**A1**	6.891	7.334	-0.443
**A2**	7.008	7.116	-0.108
**A3**	7.068	7.246	-0.178
**A4**	7.281	6.914	0.367
**A5**	7.498	6.802	0.696
**A6**	6.586	6.902	-0.316
**A7**	7.361	7.372	-0.011
**A8**	8.284	8.102	0.182
**A9**	7.910	8.148	-0.238
**A10**	7.883	7.622	0.261
**B0**	6.357	7.076	-0.719
**B1**	6.500	6.506	-0.006
**B2**	6.553	6.485	0.068
**B3**	6.710	6.591	0.119
**B4**	6.959	6.836	0.123
**B6**	6.250	6.812	-0.562
**B7**	7.134	7.220	-0.086
**B8**	7.408	7.608	-0.200
**B9**	8.108	7.405	0.703
**B10**	6.916	7.157	-0.241
**C1**	6.495	6.131	0.364
**C3**	6.655	6.517	0.138
**C5**	6.952	7.245	-0.293
**C7**	6.774	6.677	0.097
**C8**	7.063	7.432	-0.369
**C10**	8.013	7.833	0.180

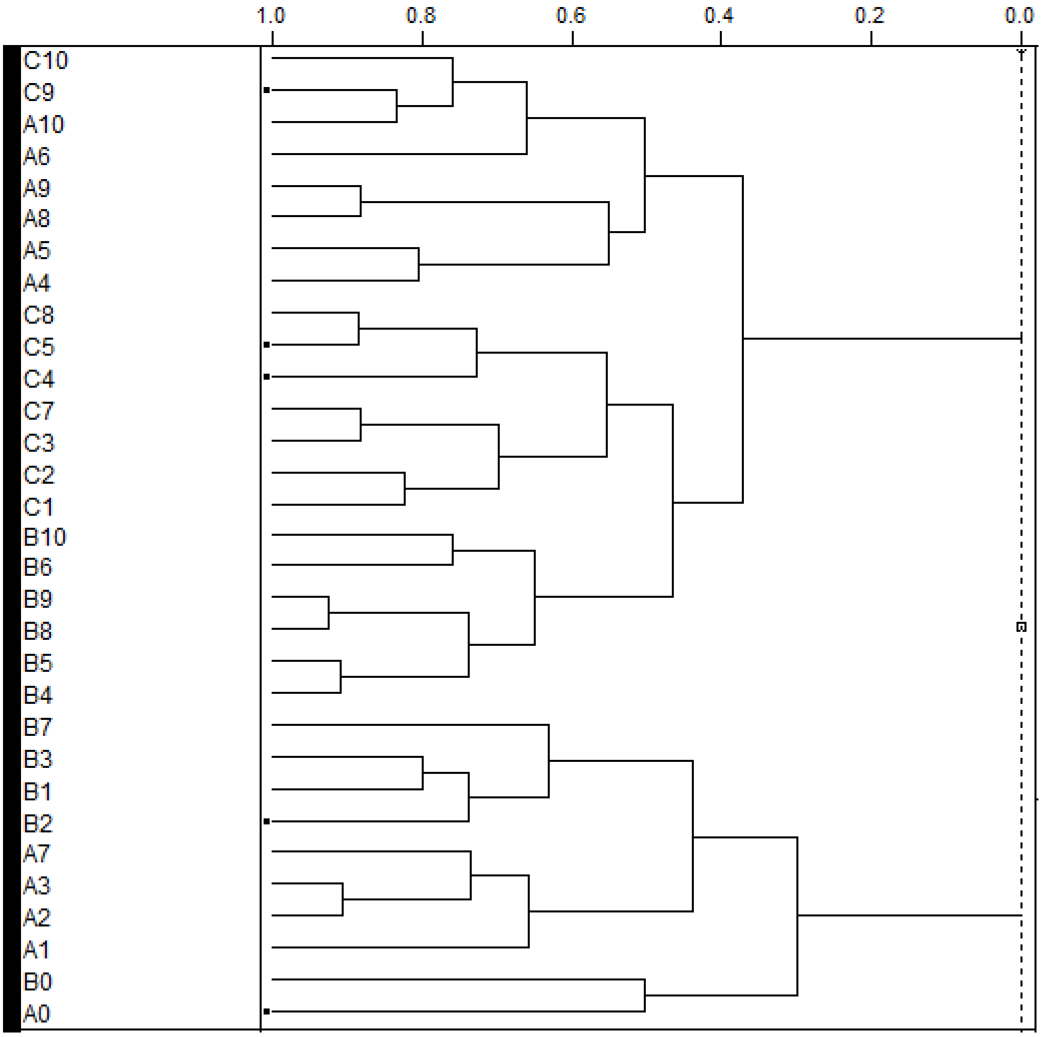

Fig. S1. Dendrogram (autoscaled data; linkage method flexible) of the data set, with the test set compounds (**A0**, **B2**, **C4**, **C5**, **C9**) highlighted (black dots). The illustration was done in the Pirouette 4 (Infometrix Inc.).

Statistical Parameters and Adopted Limits for the Evaluation of the Quality of the QSAR Model

R^2: coefficient of multiple determination of calibration[a]

$$1-\frac{\sum_i (y_i-\hat{y}_{ci})^2}{\sum_i (y_i-\bar{y})^2}$$

Expected result: R^2 > 0.6

SEC: standard deviation of calibration model[a]

$$\sqrt{\frac{\sum_i (y_i-\hat{y}_{ci})^2}{n-p-1}}$$

Expected result: as low as possible

$F_{(p,n-p-1)}$: *F*-test (with 95% confidence interval)[a]

$$\frac{\sqrt{\frac{\sum_i (y_i-\hat{y}_{ci})^2}{k}}}{\sqrt{\frac{\sum_i (y_i-\bar{y})^2}{n-p-1}}}$$

Expected result: higher than the tabulated critical value

Q^2_{LOO}: coefficient of determination of leave-one-out cross validation[b]

$$1-\frac{\sum_i (y_i-\hat{y}_{vi})^2}{\sum_i (y_i-\bar{y})^2}$$

Expected result: Q^2_{LOO} > 0.5

***SEV*: standard error of cross validation** [b]

$$\sqrt{\frac{\sum_i (y_i - \hat{y}_{vi})^2}{n}}$$

Expected result: As low as possible

***PRESS$_{val}$*: predictive residual sum of squares of validation**[b]

$$\sum_i (y_i - \hat{y}_{vi})^2$$

Expected result: higher than *SSy*

***R^2_{pred}*: coefficient of multiple determination of prediction**[c,d]

$$1 - \frac{\sum_i (y_i - \hat{y}_{ei})^2}{\sum_i (y_i - \bar{y})^2}$$

Expected result: $R^2_{pred} > 0.5$

***SEP*: standard error of prediction**[c]

$$\sqrt{\frac{\sum_i (y_i - \hat{y}_{ei})^2}{n_{ev}}}$$

Expected result: as low as possible

***ARE$_{pred}$*: average relative error of prediction**[c]

$$\frac{\frac{\sum_i |y_i - \hat{y}_{ei}|^2}{y_i} 100}{n}$$

Expected result: as low as possible

***k* and *k'*: slopes of the linear regression lines**[c]

$$k = \frac{\sum_i (y_i - \hat{y}_{ei})}{\sum_i y_{ei}} ; \quad k' = \frac{\sum_i (y_i - \hat{y}_{ei})}{\sum_i y_i}$$

Expected results: $0.85 \le k \le 1.15$; $0.85 \le k' \le 1.15$

The absolute value of the difference between the coefficient of determination between y_{obsi} and y_{evi} and the coefficient of determination between y_{evi} and y_{obsi}[c]

$$\left|R_0^2 - R'^2_0\right|$$

Expected results: $\left|R_0^2 - R'^2_0\right| < 0.3$

[a]data fit; [b]cross-validation; [c]external validation; y_i: observed pIC$_{50}$; $\bar{y}$: average observed pIC$_{50}$ for the training set; [d]for R^2_{pred}, $\bar{y}$ is the average value of observed pIC$_{50}$ for the training set without the test set; $\hat{y}_{ci}$: estimated pIC$_{50}$ in the calibration model; $\hat{y}_{vi}$: estimated pIC$_{50}$ in the cross-validation; n: number of samples in the training set; p: number of latent variables in the model; y_i: observed pIC$_{50}$; $\hat{y}_{ei}$: estimated pIC$_{50}$ in the external validation; n: number of samples in the training set; n_{ev}: number of samples in the test set; p: number of latent variables in the mode.
